# Variable Expressivity of Wolfram Syndrome in a Family with Multiple Affected Subjects

**DOI:** 10.18502/jovr.v16i4.9750

**Published:** 2021-10-25

**Authors:** Mehraban Mirrahimi, Sare Safi, Maryam Mohammadzadeh, Azadeh Doozandeh, Fatemeh Suri

**Affiliations:** ^1^Ocular Tissue Engineering Research Center, Research Institute for Ophthalmology and Vision Science, Shahid Beheshti University of Medical Sciences, Tehran, Iran; ^2^Ophthalmic Epidemiology Research Center, Research Institute for Ophthalmology and Vision Science, Shahid Beheshti University of Medical Sciences, Tehran, Iran; ^3^Ophthalmic Research Center, Research Institute for Ophthalmology and Vision Science, Shahid Beheshti University of Medical Sciences, Tehran, Iran

**Keywords:** Variable Clinical Manifestations, WFS1 Gene, Wolfram Syndrome

## Abstract

**Purpose:**

To study the genetic basis and clinical manifestations of Wolfram syndrome in a multi-affected family.

**Methods:**

Complete clinical examinations including urological, ophthalmic, neurological, and endocrinologic assessment were performed for three affected family members. Genomic DNA was extracted from peripheral blood leukocytes with salting out method and all *WFS1* exons and their flanking regions were sequenced. Candidate variation was screened for segregation in the pedigree by Sanger sequencing.

**Results:**

A known pathogenic missense mutation in *WFS1* gene (c.1885C > T which leads to p.Arg629Trp in the encoded protein) was identified in all affected individuals. Both clinical and genetic investigations confirmed Wolfram syndrome diagnosis with variable phenotypic features

**Conclusion:**

Identical mutations in the Wolfram syndrome causative gene can lead to variable manifestations of the syndrome even in the same family. Although the medical findings and clinical examination are imperative for the diagnosis of Wolfram syndrome, genetic testing is useful to confirm the diagnosis, especially in cases with possible reduced penetrance of the characteristic signs.

##  INTRODUCTION

Wolfram syndrome (WFS) also called DIDMOAD (diabetes insipidus, diabetes mellitus, optic atrophy, deafness) is a progressive neurodegenerative disease affecting multiorgan systems.^[1]^ Wolfram and Wagener first described this entity in four siblings in 1938.^[2]^ It's a kind of rare genetic disorder with a prevalence of approximately 1 per 770,000 in the United Kingdom and 1 per 100,000 in North America.^[1]^ WFS is inherited in an autosomal recessive pattern; therefore, it should be more prevalent in populations with high rates of consanguineous marriages. However, only limited cases have been reported from the Eastern Mediterranean countries where this kind of marriage is more common. There is no published data on the prevalence of the disease in Iran.^[3]^

The WFS manifestations are variable. However, juvenile-onset diabetes mellitus (DM) and optic atrophy (OA) are two characteristic features for clinical diagnosis of the syndrome that are typically exhibited at the mean age of 6 and 11 years, respectively. Other common clinical manifestations include diabetes insipidus (DI), sensorineural deafness, urinary tract involvement, and neuropsychiatric disorders.^[1]^

WFS (WFS1; OMIM 222300) is caused by a deleterious mutation in the *WFS1* gene (OMIM 606201) on chromosome 4p16, which encodes Wolframin protein; a kind of endoplasmic reticulum (ER) transmembrane glycoprotein highly expressed in pancreatic, beta insulinoma, and brain cells. The decline in Wolframin protein leads to the ER stress and malfunctioning of the cells.^[4]^ Not only more than 400 distinct mutations of the *WFS1* gene have been reported till now (http://www.hgmd.cf.ac.uk/ac/gene.php?gene=WFS1), but the less frequent phenotypic and genotypic variant of WFS (WFS2; OMIM 604928) related to mutation in *CISD2* gene (OMIM 6011507) encoding CDGSH iron sulfur domain 2 protein have also been recognized.^[5]^

In this study, we analyzed the clinical characteristics and genetic findings of a multi-affected family with variable clinical manifestations of the syndrome.

##  METHODS

The research was approved by the Ethics Committee of the Ophthalmic Research Center at Shahid Beheshti University of Medical Sciences, Tehran, Iran. All study procedures were explained to the subjects and were performed in accordance with the Declaration of Helsinki. The signed consent form was also obtained.

### Subjects and Clinical Examination

**Figure 1:**
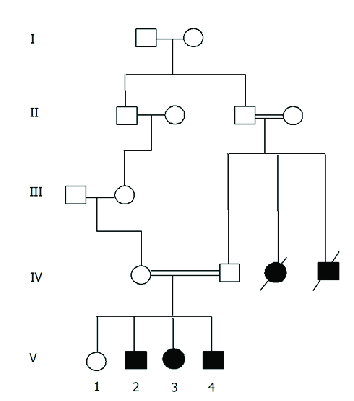
Pedigree information of the Wolfram syndrome family. Black filled shapes represent affected patients. Open shapes represent unaffected members.

Three affected siblings of Persian ethnicity from four offspring of a consanguineous marriage diagnosed with WFS referred to Torfeh Medical Center due to visual impairment were studied. Diagnosis criteria for the WFS were DM and OA unexplained by any other causes. The clinical evaluations including urological, ophthalmic, neurological, and endocrinological examinations were performed for all affected individuals. The pedigree data was gathered [Figure 1] and auditory evaluation was accomplished by pure tone audiometry, speech discrimination score, audiometric thresholds, and tympanometry. Ophthalmic evaluations including slit lamp examination, tonometry, gonioscopy, indirect ophthalmoscopy, and fundus photography were also performed. In addition, the color vision and visual field were assessed but the results were unreliable due to patients' severe visual loss. Blood samples were taken from all participants for genetic and biochemical evaluation. Brain and spinal magnetic resonance imaging (MRI) and total abdominopelvic sonography were conducted.

**Figure 2:**
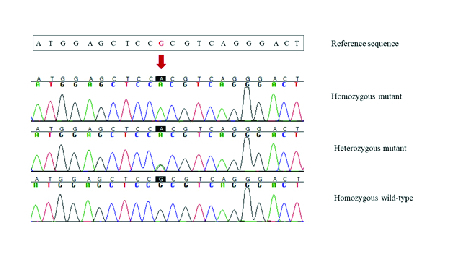
DNA sequence chromatograms of the identified *WFS1* variation in three different genotypic features in the family.

**Figure 3:**
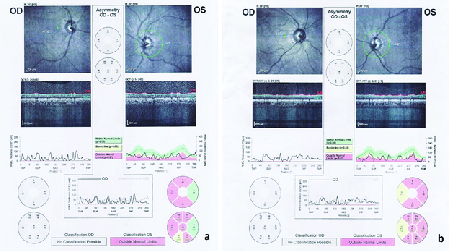
Severe nerve fiber layer loss in cases V.2 and V.3.

**Figure 4:**
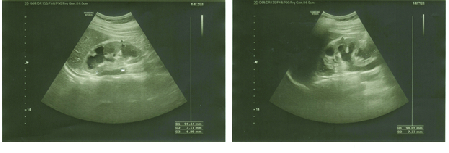
Abdominopelvic sonography in case V.3 showing severe hydronephrosis in both kidneys.

**Figure 5:**
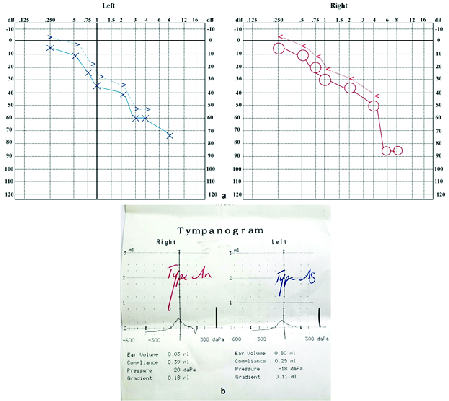
(a) Tympanogram of both ears in case V.3. (b) Audiograms of case V.3 showing sensorineural hearing loss in both ears.

**Figure 6:**
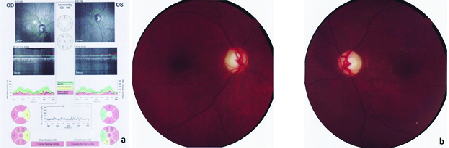
Severe optic atrophy with disc paleness in both eyes of case V.4.

### Genetic and Molecular Analysis

Genomic DNA was extracted from peripheral blood leukocytes using the standard salting out protocol. Primers were designed to amplify all exons and their flanking regions of the *WFS1 *gene using GeneRunner version 3.05 software. To validate further, we checked them for the specificity to the target template with NCBI Primer-Blast (http://www.ncbi.nlm.nih.gov/tools/primer-blast/). Polymerase chain reactions (PCR) were done and subsequently sequenced with the Sanger protocol. All PCR products were sequenced using ABI Big Dye terminator chemistry with an ABI 3730XL genetic analyzer instrument (Applied Biosystems, Foster city, CA, USA). Sequences were analyzed using Sequencher 5.0 software (Gene Codes Corporation, Ann Arbor, MI, USA). The sequencing data was assembled to their reference sequence of *WFS1* (NG_011700.1, NM_006005.3, and NP_005996.2) to find the disease causative mutation. Subsequently, the candidate causing variation was screened for segregation in all family members by Sanger sequencing.

##  RESULTS

### Genetic Results

A homozygous missense mutation (c.1885C > T) on exon 8 of *WFS1* gene that causes p.Arg629Trp in the encoded protein was detected in all three affected members of the family. The parents were heterozygous for the causative variant and the healthy sister was homozygous for the wild-type allele. Figure 2 shows DNA sequence chromatograms of c.1885 position in *WFS1 *in three different genotypic features in the members of this family. Mutation c.1885C > T has been reported to be pathogenic in patients with WFS.

### Clinical Findings

The clinical features of all participants have been represented in Table 1. The parents are second cousins once removed, having four offspring; three of them manifesting signs and symptoms of WFS [Figure 1].

**Table 1:** Clinical features of patients with wolfram syndrome


**Patient ID**	**Current age (years)**	**Gender (M/F)**	**Clinical findings**
**Case V.2**	31	M	DM, DI, OA, sensorineural hearing loss, neurogenic bladder, mild hydronephrosis
**Case V.3**	24	F	DM, DI, OA, sensorineural hearing loss, neurogenic bladder, severe hydroureteronephrosis
**Case V.4**	23	M	DM, DI, OA
DM, diabetes mellitus; DI, diabetes insipidus; OA, optic atrophy			

### Case V.2

A 31-year-old male patient was referred to the ophthalmology clinic due to visual impairment. He was diagnosed with DM at the age of four and has been on insulin therapy since then. He has been suffering from visual impairment from the age of 6. He subsequently developed DI and hearing loss at the age of 21. At the time of study, his serum HbA1C level was 7.7%. The ophthalmic evaluation showed the best-corrected visual acuity of light perception bilaterally with no nystagmus. Cataract was observed in both eyes and the intraocular pressure was 18 mmHg bilaterally. Funduscopy revealed proliferative diabetic retinopathy (PDR) in the right eye and moderate nonproliferative diabetic retinopathy (NPDR) in the left eye with no evidence of macular edema. OA with severe optic nerve head pallor was obvious in both eyes [Figure 3a]. The history and ocular examinations approved the progressive OA in the context of WFS. Abdominopelvic sonography revealed mild hydronephrosis in the right kidney with normal kidney size, normal parenchymal thickness, and echo pattern. Also, mild bladder wall thickness with trabeculation was detected. These are consistent with neurogenic bladder. Auditory tests with pure tone audiometry showed sensorineural hearing loss. Brain and lumbar spine MRIs were normal.

### Case V.3 

A 24-year-old female patient was diagnosed with DM at the age of three who has been treated with insulin NPH and regular. The presence of OA in both eyes was diagnosed at the age of 12. She developed urinary tract disorders and bladder dysfunction at the age of 12 for which she took nitrofurantoin twice daily for prophylaxis. The diagnosis of DI was confirmed with clinical signs and symptoms, laboratory tests and water deprivation test abdominopelvic sonography revealed severe hydroureteronephrosis with cortical loss in both kidneys in addition to irregularity and increment in bladder wall thickness that is consistent with neurogenic bladder [Figure 4]. Her visual acuity was finger counting at 1–2 m. Intraocular pressure was 19 mmHg in both eyes. Fundus examination revealed bilateral severe OA and nerve fiber loss [Figure 3b]. The audiometry showed sensorineural hearing loss in both ears which were moderate at 3000 Hz and severe at 6000 Hz test tone [Figure 5a]. Tympanometry presented type A ${}_{n}$ in right and type A ${}_{s}$ in left ear which suggested the reduced tympanic membrane mobility in the left ear [Figure 5b]. Brain and lumbar spine MRI revealed normal findings.

### Case V.4

A 23-year-old man was diagnosed with insulin-dependent DM at the age of four which has been treated with insulin NPH and regular since the diagnosis and subsequently was diagnosed with DI. He subsequently developed visual impairment and OA at the age of 11. His serum HbA1c level was 9.4% which represented uncontrolled DM. His best-corrected visual acuity was finger counting at 20 cm in the right eye and hand motion in the left eye. He underwent phacoemulsification and intraocular lens implantation in both eyes. Intraocular pressure was 18 mmHg bilaterally. On funduscopic examination, there was severe OA [Figure 6]. His abdominopelvic sonography reported normal kidney sizes with normal parenchymal thickness and echo patterns. Neither hydronephrosis nor stone or occupying lesion was detected. Additionally, bladder wall thickness and shape was normal without any evidence of trabeculation or neurogenic bladder. He occasionally reported urological symptoms which was proved by the normal results of his abdominopelvic sonography. He had no complaint of auditory loss and his auditory tests were normal.

##  DISCUSSION

WFS is a rare progressive neurodegenerative disorder characterized by OA and DM at childhood which is inherited in an autosomal recessive pattern.^[6]^ Patients affected by this syndrome can manifest different signs and symptoms of the disease, for example, DI, sensorineural hearing loss, and neurological signs such as ataxia, and neurogenic bladder in combination with DM or OA.^[6]^ According to clinical history and physical examination, the differential diagnosis could include other causes of progressive neurodegeneration like mitochondrial disorders, mutant *WFS1* gene-induced deafness, autosomal dominant OA, Friedreich ataxia, Bardet–Biedl syndrome, and Alström syndrome.^[7]^ Among this wide spectrum of clinical manifestations, juvenile-onset DM and OA are both initial and fundamental features of WFS.^[8]^ In this study, we reported the clinical and genetic characteristics of three patients with WFS from a consanguineous marriage. So far, a wide range of *WFS1* gene variants has been recognized as cause of the disease.^[4]^ Most of these mutations result in loss of function protein expression which is responsible for Wolframin protein inactivation and consequent disease manifestations; however, the exact genotype–phenotype correlation in WFS-affected patients is not well understood.^[9,10]^ In this study, we detected a known homozygous pathogenic mutation (c.1885C > T) in exon 8 of *WFS1* gene in all the three affected patients that has been reported in other studies as cause of the disease.^[11,12,13]^ Furthermore, we discovered that both parents were heterozygous for the causative variant who were healthy and the youngest sister of the family was homozygous for the wild-type allele. Therefore, not only the clinical diagnostic criteria but also the genetic analysis in all three cases was consistent with WFS. DM usually occurs during the first decade of patients' lives.^[11]^ In our study, all of our cases suffered from juvenile-onset DM with the average age at onset of 3.8 years (ranging from 3.5 to 4 years). While diabetes ketoacidosis (DKA) may occur as the presenting features of the syndrome,^[14]^ none of our cases have experienced any episodes of DKA or severe hypoglycemia yet. As previously mentioned, Wolframin protein is found in the ER of Langerhans β-cells and loss of function mutations of the *WFS1* gene can lead to Wolframin protein dysfunction which consequently leads to ER stress.^[15]^ This phenomenon can lead to non-autoimmune-mediated/induced destruction of β-cells and subsequently, DM occurs. This pathophysiology is consistent with the early onset DM in WFS-affected patients in comparison with autoimmune-mediated destruction of β-cells in type 1 DM which is triggered later during life.^[16]^ Patients with WFS will progressively develop ophthalmic complications in consequence of DM, that is, diabetic retinopathy and cataract.^[8]^ We discovered diabetic retinopathy and cataract in both eyes in one of the cases (Case V.2) which is justified by longer duration of disease (27 years) in comparison with other participants. In addition to DM, OA is another characteristic feature of the syndrome which usually develops after DM at the mean age of 11 years.^[1,11]^ According to OA pathogenesis, *WFS1* gene mutation reduces the retinal ganglion cells survival which leads to retinal axon layer atrophy and optic nerve shrinkage. The latter will also result in progressive OA.^[8]^ All of our cases had symptoms and signs of OA after the diagnosis of DM. The mean age at the onset of OA in our cases was 9.6 years.

Other studies have reported hearing impairment in 60% of WFS cases at the mean age of 16 years.^[17]^ Auditory features of this hearing impairment were compatible with sensorineural hearing loss in mid to high frequencies. The pathophysiology of this kind of hearing loss could be the result of dysfunction in cochlear neurons, vestibular nerve fibers of cranial nerve VIII, and the central nervous system (including brain stem and inferior colliculus).^[18]^ In our study, we detected a sensorineural hearing loss in high frequencies confirmed by an audiometry in two of our cases (Cases V.2 and V.3).

Urinary tract involvement is one of the signs of the WFS. This manifestation mainly has a primary feature rather than being secondary to DM, DI, or WFS-induced myelopathy and could be present independent of other features.^[19]^ In our study, we have found evidence of neurogenic bladder, that is, hydronephrosis and bladder wall trabeculation in two cases (Cases V.2 and V.3) which was mild in the former and severe in the latter. These findings could be a result of diabetic-induced peripheral neuropathy,^[20]^ however, since neither of our cases presented any signs and symptoms of autonomic or peripheral neuropathy, this finding will not be probable. In our study, we found no signs and symptoms of neurological complications neither in physical examination nor in MRI images of the brain and spine.

In conclusion, our study showed that an identical mutation in *WFS1* gene can represent variable severity of clinical features of the disease even in the same family. More studies on age- and sex-matched cases may shed light on reasons behind this variable clinical presentation and the role of genetic background and environmental factors. Additionally, further studies exploring different *WFS1* mutations will improve our knowledge about genotype–phenotype correlations of this syndrome.

##  Financial Support and Sponsorship

None.

##  Conflicts of Interest

There are no conflicts of interest.
